# Bill Sykes's Son
*The Heredity of Crime. By Thomas M. Dolan, M.D., F.R.C.S.Edin. A Lecture delivered at the Halifax Literary and Philosophical Society, January, 1890. Reprinted from the *Provincial Medical Journal*. May, 1891.


**Published:** 1892-07-09

**Authors:** 


### Bill Sykes's Son.*

The persistence with which some advocates of the doctrine of heredity have pushed their theory into all branches of sociology has produced an inevitable reaction. It is felt that to say that a man is his father's son is not a full explanation of any mental and moral peculiarities he may show; and that to assume the constant and unvarying reproduction of parental characteristics from generation to generation is not only to deny many obvious facts of human life, but to give way to a fatalism which paralyses all attempt at progress. Dr. Lombroso, of Turin, the well-known specialist in criminal anthropology, is an ardent believer in a criminal type, so unvarying in aspect that it labels all who bear it as actual or possible law-breakers. Dr. Dolan, of Halifax, while admitting that a temperament may be vaguely indicated by a type of face, holds that such indications may be counteracted by environment, education, and the development of the moral nature. To speak theologically, they are the advocates respectively of predestination and free-will—each, perhaps, preaching a part of the truth. That children do inherit tendencies of mind as well as characteristics of person from their parents is as undeniable as that every man, by the exercise of his will-power can more or less improve the quality of his nature. We are more than automata, dancing at the bidding of heredity, or fate, or any other name by which we may choose to label an outside power; less than gods, able "to will and to do of our good pleasure."

"Why," asks Dr. Dolan, "do not a drunkard's children all and always become intemperate? The sons often do; the daughters usually escape. Is not this because the sons are in the way of temptation, and the daughters are not?"

Yes; because the sons, being tempted, have that predisposition which tends to make them yield; and though the daughters, free from that temptation, may not so yield, they may instead be nervous, hysterical, delicate in health, and capricious in will. Circumstances may alter the form such a predisposition may take, and modify its intensity so much as, in two or three generations, given all things favourable, practically to eradicate it. But this is not the same thing as saying that the drunkard's child has as good a chance in life, either morally or physically, as the offspring of the self-controlled and temperate man. If Bill Sykes's son be taken away from the filthy court and its degrading associations, and surrounded by wholesome influences, given pure air and sufficient nourishing food—things not to be neglected in aiming at moral results—he has a chance of not growing up to be a thief and a murderer, like his father; but it is hardly to be expected that he will grow up to be a distinguished philanthropist. Nay, he has that in his inmost nature which will make him an easy victim to any temptation that may beset him, unless his training has included the careful education of his will.

There is no doubt that statements made from the dispassionate standpoint of the scientific observer without a thought of harm, may, when carried by a self-excusing weakling into the domain of morals, work incalculable harm. And it is impossible to keep any scientific fact from being so applied; man being, by the constitution of his nature, a self-conscious being, unable to look at any subject without asking, "How does this affect me?" Therefore, any attack on the supremacy of the will is a distinct temptation to cowardice, laziness, and every graver evil that springs from living by impulse, not by principle.

"How free we seem, how fettered fast we are," moans Andrea del Sarto; and excuses failure and dishonesty by saying, "All is as God overrules!"

The tendency of our age has been to put all things on a physiological basis, but physiologists themselves would admit—though, perhaps, their less thoughtful followers would not—that when their last word is said, their last explanation offered, the *ego*, the will, the individuality remains unexplained. The theory of heredity belongs to the physiological side of man, to size and quality of brain, to nervous conditions inherited not only from parents, but from ancestors more remote; but beyond these lies the will, susceptible to moral influences and spiritual appeals. Not, indeed, equally susceptible in all. A life of self-indulgence and vice may almost kill this spark that most closely links us with the Divine—to save Bill Sykes is, humanly speaking, hopeless. But every new birth is a new potentiality—the will is born again, feeble, perhaps degenerate, but containing still some potentialities. Something—not, indeed, something great, but something honest and harmless may be made of Bill Sykes's son.

* The Heredity of Crime. By Thomas M. Dolan, M.D., F.R.C.S.Edin. A Lecture delivered at the Halifax Literary and Philosophical Society, January, 1890. Reprinted from the *Provincial Medical Journal*, May, 1891.

